# Role of Ion Dehydration in Ion–Ion Selectivity of Dense Membranes

**DOI:** 10.1021/acs.est.5c04303

**Published:** 2025-08-19

**Authors:** Alexander Ershov, Hengyu Xu, Ying Li, Tiezheng Tong, Razi Epsztein

**Affiliations:** † Faculty of Civil and Environmental Engineering, 26747 Technion—Israel Institute of Technology, Haifa 32000, Israel; ‡ Department of Mechanical Engineering, 5228 University of Wisconsin-Madison, Madison, Wisconsin 53706, United States; § School of Sustainable Engineering and the Built Environment, Arizona State University, Tempe, Arizona 85287, United States

**Keywords:** nanofiltration membranes, polyamide membranes, dense membranes, ion−ion selectivity, ion dehydration, energy barrier, compensatory interactions

## Abstract

Fabricating polymeric membranes with ion-specific selectivity has been targeted in recent years to address the growing challenges of water and resource scarcity. Inspired by discoveries of the selectivity mechanisms in biological channels, ion dehydration has been increasingly recognized as a key phenomenon governing the transport and selectivity in dense polymeric membranes and other synthetic nanochannels. However, understanding the molecular details of this phenomenon and leveraging and controlling it to increase the selectivity between ions in state-of-the-art membranes remain elusive. In this Perspective, we discuss the foundations of ion dehydration and explore opportunities to study and leverage this phenomenon for improving ion–ion selectivity in membranes. We first introduce the fundamentals and measurements of ion’s hydration properties in solution, distinguishing between static and dynamic hydration properties. Next, we discuss simulation and experimental techniques to study ion dehydration under confinement, highlighting critical knowledge gaps that impede our understanding of this phenomenon. We then discuss effects of ion dehydration on the energy landscape of ion transport and analyze attempts in the literature to improve ion selectivity by promoting dehydration of specific ions. We conclude by proposing research directions to enhance our understanding of ion dehydration and fabricate sustainable and robust membranes with ion-specific selectivity.

## Graphical abstract

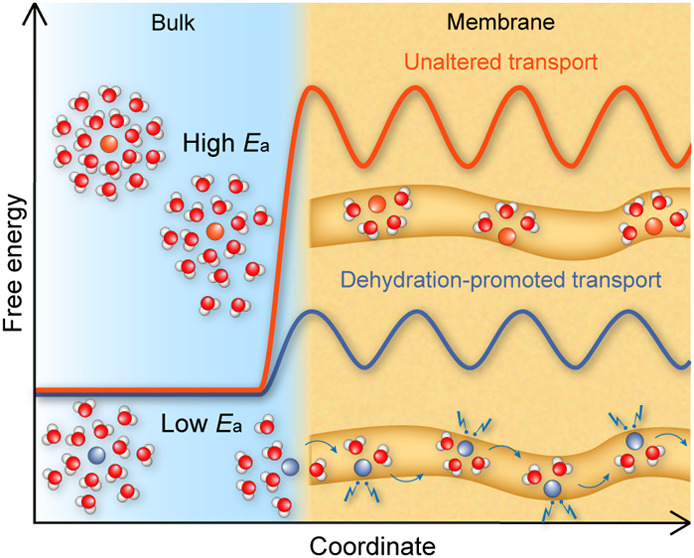

## Introduction

During the past several years, growing scientific efforts have been directed to understand and improve the selectivity between solutes in dense polymeric membranes, such as nanofiltration and ion-exchange membranes.^1–14^ This membrane ability to distinguish between different solutes is necessary to achieve fit-for-purpose and more sustainable water treatment and enable recovery of valuable elements from complex streams.^1,15^ However, compared to the separation of salt from water, which is the key requirement in desalination processes,^16–19^ achieving high and sufficient separation between solutes using state-of-the-art polymeric membranes remains a daunting challenge.^1,20^

As many of the target species in water treatment and resource recovery are inorganic ions, it is imperative to understand the mechanisms that govern ion–ion selectivity, which predominantly involve steric (size) and Donnan (charge) effects.^21–28^ Dielectric exclusion, which refers to hindering ion transition from a high-dielectric medium (*i.e.*, water) to a low-dielectric medium (*i.e.*, the membrane), is an additional mechanism that is often suggested to explain the selectivity between ions based on their different solvation energy.^29,30^ While these mechanisms can promote selectivity between ions, the resultant selectivity is frequently low and insufficient for practical separation. Specifically, differentiating between similarly sized and charged ions (*e.g.*, two monovalent ions) requires leveraging mechanisms beyond the above-mentioned mechanisms.^31–36^

Within the last two decades, research efforts using molecular dynamics simulations^37–40^ and experimental permeation tests^41–49^ have suggested that the ion’s hydration shell in water can be partially removed or rearranged during ion permeation through pores that are smaller than the hydrated size of the ion. As these variations are ion-specific, ion dehydration opens opportunities to achieve selectivity between similarly sized and charged ions.^37,50,51^ Notably, ion dehydration is a key step to obtain selectivity in ion-selective biological channels, where ion-specific binding sites serve as surrogate water and compensate for the energetic cost of dehydration by creating attractive (stabilizing) interactions with the target dehydrated ion.^52–54^

Despite the growing insight into ion dehydration, a robust molecular-level understanding of this phenomenon remains incomplete, particularly in disordered, flexible polymeric environments, due to the lack of direct experimental evidence.^41,55^ As a result, many questions remain open regarding the specific molecular details that characterize this phenomenon in polymeric membranes, its actual location, and the feasibility to utilize it to improve the selectivity between ions. In this Perspective, we first introduce the origin and fundamentals of ion hydration in water and its characterization using traditional methods and state-of-the-art molecular simulation techniques. Next, we discuss simulation and experimental evidence of ion dehydration in membranes and channels, highlighting prominent knowledge gaps and uncertainties that require further investigation. We conclude by interpreting dehydration-originated ion–ion selectivity in literature and proposing sustainable avenues to study ion dehydration in membranes and synthesize polymeric membranes that promote dehydration of ions in a more selective manner.

## Ion Hydration in Water

### Origin of Ion Hydration

Although water molecules (H_2_O) are electrically neutral, their positive and negative charge centers do not coincide due to their bent geometry, which features a H–O–H bond angle of approximately 104.5°.^56^ This asymmetry leads to a dipole moment of 1.85 D,^57^ resulting in a partial negative pole (δ^–^) near the oxygen atom and a partial positive pole (δ^+^) near the hydrogen atoms (Figure 1a). Due to this dipole, charged ions in solution attract the oppositely charged poles of nearby water molecules, constraining them to form the ion’s hydration shell (Figure 1b). Since electrostatic interactions are the major contributors to ion hydration,^58^ the structure of the hydration shell primarily depends on two electric-related factors. The first factor is the surface charge density of ions, which indicates that each ion possesses a unique hydration shell structure depending on its charge and bare size. The second factor is the local electric field induced by other polar solutes existing in the solution. Since the hydration shell impacts both the chemical and physical properties of ions in solution,^59,60^ quantitatively investigating ion hydration structures is crucial for understanding their broader implications.

**Figure 1:**
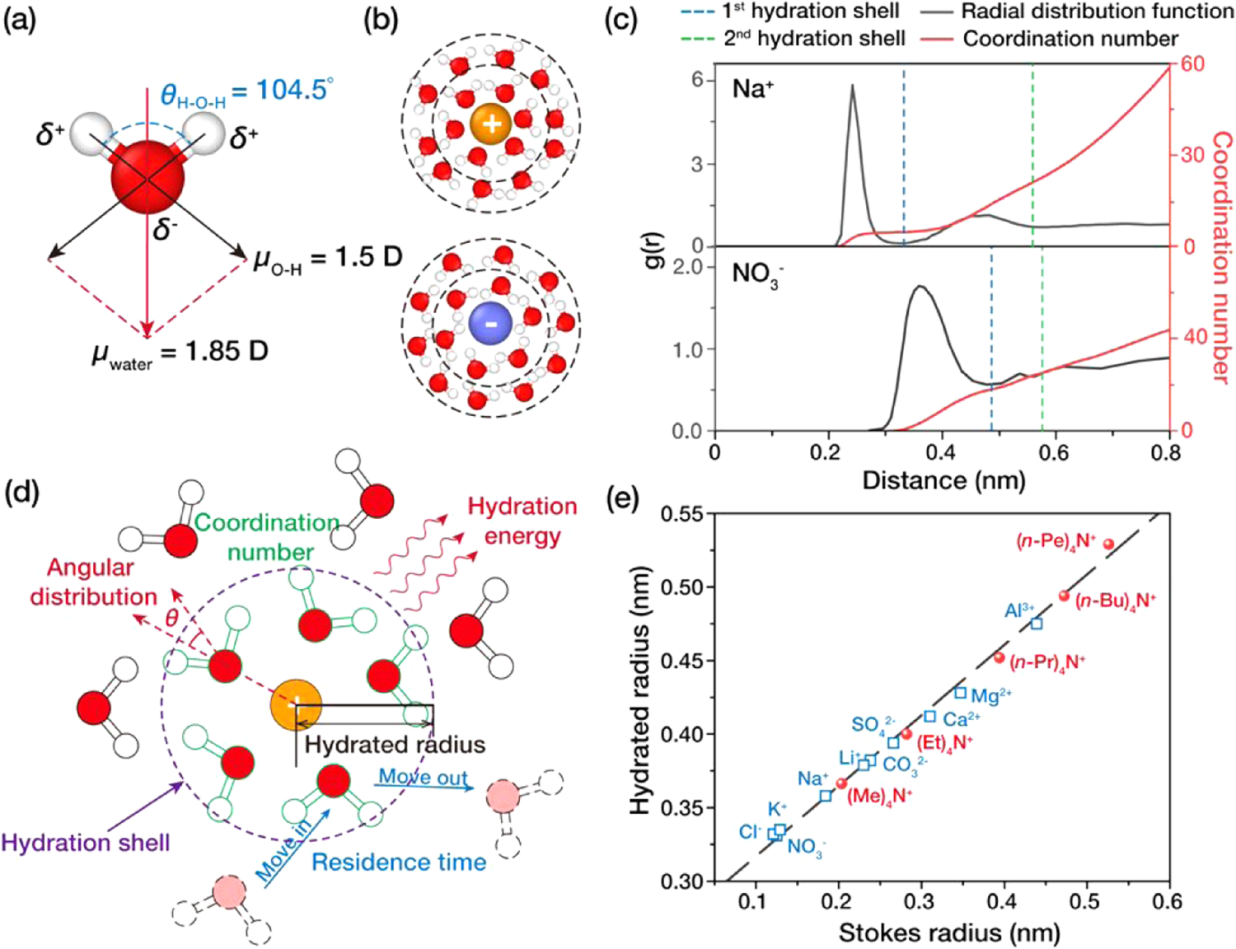
Structure and measurement of ion hydration. (a) Structure and dipole moment of a water molecule. (b) Hydration structure of cations and anions. (c) Radial distribution function (RDF) of water around Na^+^ and NO_3_^–^ ions and their corresponding coordination number, taken from refs 61 and 62, respectively. (d) Schematic illustration showing the characteristics of ion hydration, including the hydration shell, hydrated radius, coordination number, angular distribution, residence time, and hydration energy. (e) Relationship between the Stokes radius and hydrated radius of major ions. The data shown was taken from ref 63. Red dots represent tetraalkylammonium ions, which are used to establish the calibration curve.

### Measurement of Ion Hydration

Since water molecules in the hydration shell are constrained by ions through electrostatic interactions, their density in the proximity of ions differs from that in a more distant location from the ions in the bulk phase. This feature suggests that an efficient way to measure ion hydration is by determining the radial density profile of water around the ion, commonly represented by the radial distribution function (RDF). As shown in Figure 1c, the RDF curve typically exhibits multiple density peaks within the hydration shell,^64^ indicating that the electrostatic interaction extends beyond the size of individual water molecules, forming a multilayered hydration shell around the ion. Therefore, the hydration shell can be divided into multiple subcoordination shells (Figure 1c), and the amount of water molecules within each shell represents the corresponding coordination number.

Water molecules in the multilayered hydration shell continuously exchange with the bulk solvent, particularly those that interact weakly with the central ion *via* electrostatic forces. Within this framework, ion hydration properties are broadly categorized into static (structural) and dynamic (hydrodynamic) properties. Static properties, typically obtained from RDFs, describe the spatial organization of water around the ion, including features such as coordination shell size and angular distribution of surrounding water molecules. Instead, dynamic properties pertain to ion transport and capture how the surrounding water molecules influence ion mobility, such as hydrated radius and residence time of hydrated water molecules (Figure 1d). Although the numerical values of static and dynamic hydration properties may coincide for certain ions (*e.g.*, the static and dynamic coordination numbers of sodium are 4–6 ^65^ and 4.6,^65^ respectively), these two concepts are fundamentally distinct in both origin and physical meaning.

Notably, RDF results indicate that the size of the hydration shell falls within the subnano scale, making it challenging to directly measure the characteristics of ion hydration. Before the establishment of advanced experimental and simulation techniques, measurements of these characteristics were obtained by indirect methods, such as correlating the kinetic behavior of ions in solution to their size with traditional models. Specifically, Nightingale estimated the effective hydrated radii for major cations and anions by establishing a calibration curve that correlates the Stokes’ law radii, which is calculated from the limiting equivalent conductance of ions, to the effective hydrated radii of ions using tetraalkylammonium ions whose radii in solution are known (Figure 1e).^63^

Although the values of ion’s hydrated radii reported by Nightingale have been used in many studies (Table S1),^2,21,65–67^ some literatures have reported differing values for these radii. For example, the hydrated radius of the nitrate ion reported by Nightingale is 0.332 nm.^63^ However, as shown in Figure 1c, the size of the first hydration shell of nitrate obtained from both molecular dynamics (MD) simulations^37,68^ and density functional theory calculations^62^ is approximately 0.5 nm. This discrepancy is attributed to the conflation of static and dynamic hydration properties. Static properties, typically characterized by RDF, are independent of the strength of electrostatic interaction between ions and surrounding water molecules. In contrast, only strongly bound water molecules contribute to an ion’s dynamic hydration properties.^69^ Therefore, it is essential to clearly distinguish dynamic from static dynamic hydration properties in all theoretical, experimental, and simulation studies.

Nowadays, with the assistance of state-of-the-art experimental techniques, scattering (*e.g.*, X-ray diffraction) and spectroscopic (*e.g.*, nuclear magnetic resonance) approaches are widely employed in measuring ion hydration. The scattering methods can directly detect the structure factor of ion hydration and then convert it to RDF through Fourier transform; meanwhile, the spectroscopic approaches are able to estimate the ion hydration characteristics through chemical shifts, nuclear Overhauser effect (NOE), and other parameters.^70^ The hydrodynamic properties can be evaluated according to the electrostriction,^71^ entropy analysis,^72^ or dielectric relaxation spectroscopy^73^ principles. Although great progress has been made in this field, most of these methods become ineffective under complex conditions, such as in the presence of an electric or magnetic field or within a confined environment.^74,75^

Simulation methods offer unique advantages that overcome such limitations. Specifically, MD simulations are the most widely used techniques to study ion hydration because of their proper balance between computational accuracy and efficiency. MD simulations iteratively update the position and velocity of each atom on the basis of potential energy distribution and thermostat controller, enabling the capture of all static, dynamic, and energetic properties of ion hydration.^76–78^ Comparisons between experiments and MD simulations have shown excellent agreement when appropriate potential field parameters are applied.^70,76,79^ Moreover, advancements in computational power have further facilitated the role of MD simulations in quantifying ion hydration. Therefore, by using a combination of experimental and computational approaches, ion hydration properties can be precisely characterized.

One noticeable observation of hydration shells is that both hydrated radius and hydration energy of major ions decrease with increasing ionic radius and decreasing ionic charge (Table S1, example for cations).^58,63,76^ This trend is attributed to the reduction in surface charge density for ions with larger radii and/or lower charges, weaking their electrostatic attraction to water molecules. Notably, as ions diffuse in solution, their hydration shells move along with them, reaching a dynamic equilibrium that largely preserves the ion hydration structure. However, under extreme conditions, such as strong electric fields^80^ or diffusion under confinement,^37,81^ the coordination number decreases; that is, the ion gives up some of its surrounding water molecules—a phenomenon commonly known as partial dehydration, as we discuss in the next section.

## Ion Dehydration in Membranes and Nanopores

### Shell Rearrangement under Confinement

The ionic hydration shell significantly increases the ion size,^63^ hindering its transport through membranes with pores of smaller or similar dimensions.^38,41,82–88^ Therefore, when ions partition from water to a membrane with narrow pores (or voids) as part of their transmembrane permeation, some modification in the hydration shell may be needed for the ions to reduce their size (or cross-sectional area) and fit within the membrane pore;^37,84^ such modification can affect both the static and dynamic hydration properties of the ion. Since this ability of ions to rearrange their hydration shell is ion-specific, dehydration by itself can promote ion–ion selectivity.^1,50,86^ Notably, when ions dehydrate or rearrange their shell, they can more closely interact with chemical groups inside the pore;^89–95^ depending on the nature of such ion-specific interactions, the partitioning and following intrapore diffusion of ions can be further tuned to enhance the ion–ion selectivity, as we discuss later.

While ion dehydration has been demonstrated using X-ray crystallography and established as a key mechanism to achieve selectivity in biological channels during the late 1990s and early 2000s,^52–54,96–99^ it has started attracting growing attention by the membrane and nanofluidic communities a few years later following several pioneering simulation studies on the topic.^37,51,100,101^ In these studies, it was shown that the ability of ions to undergo dehydration governs their permeability and that this ability can be directly connected to the energy barrier associated with the ion transport. Such dehydration-related energy barriers to ion transport through membranes and pores, which highlight the role of thermal energy in enabling dehydration, vary significantly among different scenarios depending on the ionic (*e.g.*, hydrated radius and hydration energy) and membrane (*e.g.*, pore size and charge) properties.^89,101^ In the following section, we discuss major findings on ion dehydration in literature and highlight critical knowledge gaps and limitations that hinder our understanding of ion transport.

### Key Findings and Knowledge Gaps in Ion Dehydration

Molecular dynamics simulations provide the most compelling evidence of ion dehydration in membranes and narrow pores.^102^ While the picture obtained in this case is based on theoretical calculations of the potential energy landscape from a set of existing parameters describing the forces between atoms (*i.e.*, force field), these simulations provide strong indication that ion dehydration is energetically feasible under practical conditions, and that trans-pore permeation cannot take place without it in certain cases. The occurrence and extent of ion dehydration is most commonly demonstrated by generating the free energy profile together with calculation of the coordination number of the ions along the transport coordinate.^37–39,51,85,86,101,103,104^ Results from these calculations typically show a free energy barrier associated with decrease in the coordination number when ions move from the solution into the pore (Figure 2a,b), establishing ion dehydration as the main contributor to the free energy barrier to transport through narrow pores.

**Figure 2:**
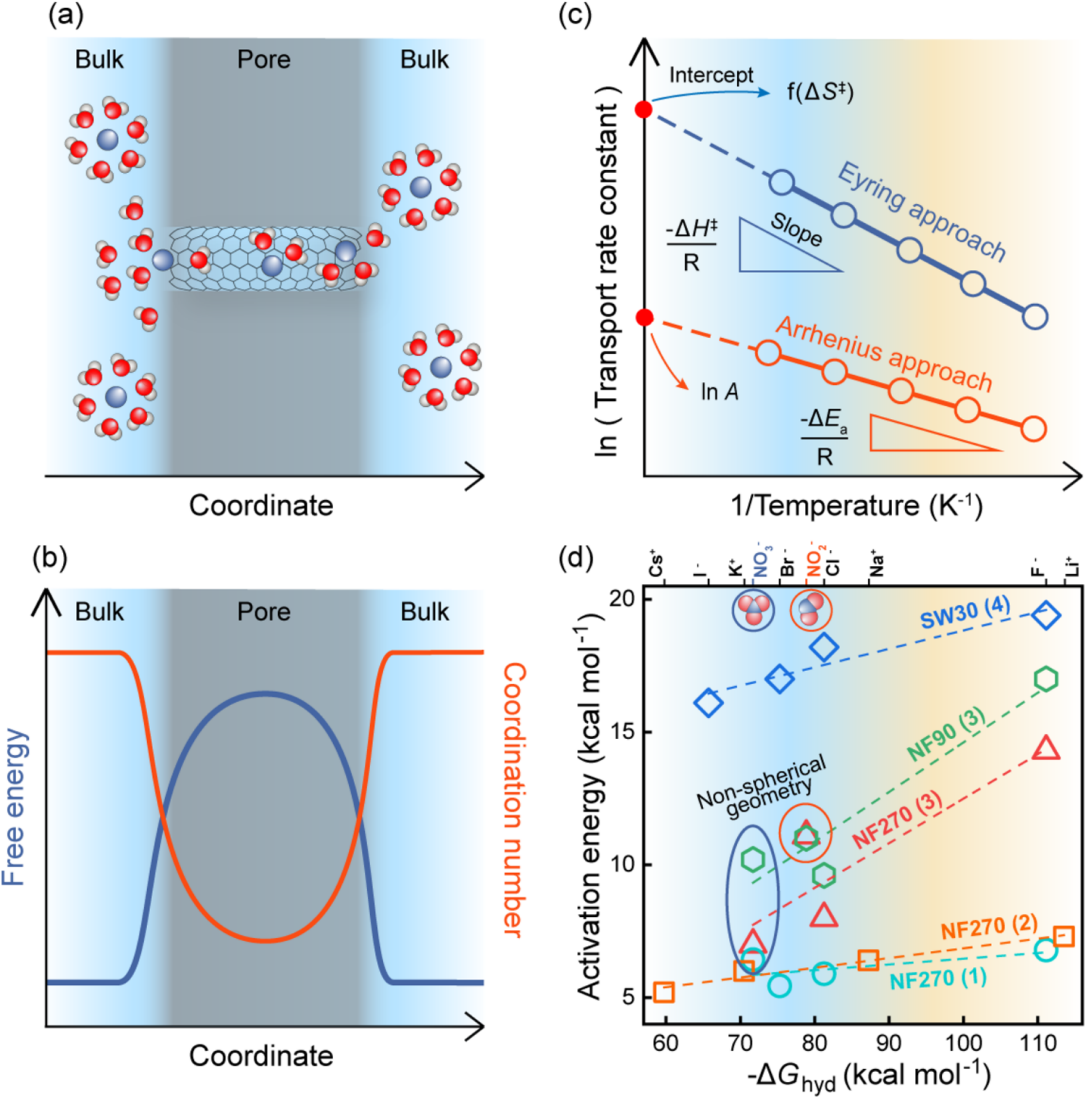
Exploring ion dehydration under confinement. (a, b) Schematic of MD simulations of ion transport in narrow pores. (a) Schematic representation of ion transport through a carbon nanotube. (b) Variation in free energy and coordination number of an ion as it moves through a confined environment. (c, d) Experimental determination of energy barriers for ion permeation. (c) Experimental energy barrier calculations using the Arrhenius (orange) and Eyring (blue) approaches, where the transport rate constant is plotted as a function of the temperature reciprocal. In the Arrhenius approach, the activation energy (−Δ*E*_a_) is determined from the slope and the pre-exponent factor (*A*)—from the intercept with *Y*-axis. In the Eyring approach, the enthalpic barrier (−Δ*H* ^‡^) is determined from the slope and entropic barrier (−*T*Δ*S* ^‡^)—from the intercept with *Y*-axis. (d) Correlation between hydration free energy and activation energy for different membranes, determined from refs 105­(1),^106^­(2),^41^­(3), and^43^­(4).

Using this method, the roles of fundamental pore and ionic properties in promoting ion dehydration were studied. For example, multiple simulations showed that smaller pore size and higher hydration energy of the transported ions result in higher free energy barrier to ion transport due to more hindered dehydration.^37,38,85,86,100,101^ Such varying ability of ions possessing different hydration properties to undergo dehydration establishes an intrinsic ion–ion selectivity trend in membranes and nanopores and lays the foundation for further improvement (or reversal) of such selectivity by adjusting the pore structure and chemistry to promote the dehydration of specific ions, as we discuss later.^1,50,86,93,94^ Despite the intriguing success of MD simulations in demonstrating and studying ion dehydration, these simulations are still limited in their ability to model ion transport in complex and more realistic membrane systems with pore size distribution, heterogeneous structure, and large thickness that requires impractically high computational time.^79,102,107–109^

Due to the difficulty to experimentally probe ion dehydration inside pores (*i.e.*, during permeation), most experimental work only indirectly supports ion dehydration based on correlations between ionic hydration energy and measured energy barriers to transport by quantifying the ion transport rate at different temperatures according to the Arrhenius^35,41,43,44,83,89,105,106,110–114^ or Eyring^34,42,55,115–119^ approach (Figure 2c). Specifically, it was shown that the Arrhenius activation energies or Eyring’s enthalpic barriers to transport through dense membranes increase for more strongly hydrated ions or smaller pores,^34,41,42,44,55,83,105,110,112^ supporting MD simulations of transport through artificial pores that highlight dehydration’s major role in the energy barriers to transport (Figure 2d).^37^ While the two approaches are based on the measurement of ion permeability at different temperatures, the Eyring approach provides more specific and detailed picture of the enthalpic and entropic barriers associated with ion transport in the membrane. However, unlike simulated barriers by MD, experimentally measured activation energies in polymeric membranes are effective properties that do not represent a single or specific barrier in the transport and are affected by contribution from multiple hindering mechanisms, which can often lead to inaccurate or biased conclusions regarding the mechanisms involved.^120^ In addition, these measurements present substantial uncertainties due their high sensitivity and intrinsic methodological constraints.^34,116,121,122^ Due to these challenges, measured barriers are sometimes counterintuitive,^106,116^ unphysically high^123^ or low,^35,124^ and inconsistent among different studies—limitations that can be amplified by the heterogeneity of polymeric membranes^120,125–127^ and interfering phenomena that are hard to accurately account for, such as concentration polarization and polymer relaxation.^44,128^

A more recently proposed experimental method to probe ion dehydration in polymeric membranes in real time is *in situ* liquid time-of-flight secondary ion mass spectrometry (ToF-SIMS). In this system, the transport of water and ion molecules across a polyamide membrane is driven by applying an ultrahigh vacuum at the membrane surface, with a confined liquid-vacuum interface being exposed to a Bi_3_^+^ beam. The permeate of the membrane is then bombarded by Bi_3_^+^ to generate secondary ions that are subject to mass spectrum analysis. To our knowledge, this approach represents the only method reported to experimentally analyze ion dehydration across polyamide membranes commonly used in nanofiltration and reverse osmosis applications. However, it is limited to the detection of the ionic hydration adjacent to the pore exit, rather than inside the pore.^55,83,129^

Compared to the limited experimental characterization of ion dehydration in polymeric membranes, multiple techniques were applied to study the state of ion hydration in confined (nonpolymeric) nanochannels including X-ray diffraction (XRD),^130^ extended X-ray absorption fine structure (EXAFS),^131^ and nuclear magnetic resonance (NMR),^132^ which can be designed *in*- or *ex- situ*. However, these techniques have been rarely used to characterize ion hydration state and ion dehydration in polymeric membranes, likely due to the lack of sufficient temporal and spatial resolution. Recently, EXAFS was applied to characterize the coordination number and (indirectly) the hydration number of vanadyl cations within polymeric ion-exchange membranes,^133^ but its applications to ion dehydration within dense polyamide membranes have not been reported in the literature. While molecular simulations and experimental energy barriers establish dehydration as a key phenomenon in transmembrane permeation, the exact physical way it manifests under different conditions is still challenging to describe due to the limitations of existing measurements described above, rendering the picture of transport in membranes partially speculative. Advancements in measurement tools are therefore imperative to probe and study partially dehydrated ions within the pore, making the picture of ion transport under confinement more certain.

## Dehydration-Promoted Ion–Ion Selectivity

### Principles of Ion Dehydration in Establishing Ion–Ion Selectivity

Conventional selectivity mechanisms, such as size and charge exclusion, often fail to fully explain differences in ion transport.^23,49,55,133^ In many of these cases, ion dehydration—alone or in combination with other recently proposed mechanisms—plays^30,134,135^ a crucial role in governing the ion selectivity.^35,87^ Most notably, dehydration can explain the selectivity in particular scenarios where ions have similar charge and hydrated size but substantially different hydration energies,^37,53,136^ such as K^+^ *vs* Na^+^ or Cl^–^ *vs* F^–^. In these cases, despite comparable hydrated radii,^63^ variations in the free energy of hydration (Δ*G*_hyd_)^137^ can lead to ion–ion selectivity due to ion-specific energy penalties for dehydration (Figure 3a).^43^ These dehydration-related energy barriers for ion transport can be tuned,^138^ offering a pathway to enhance ion–ion selectivity, as we discuss below.

**Figure 3:**
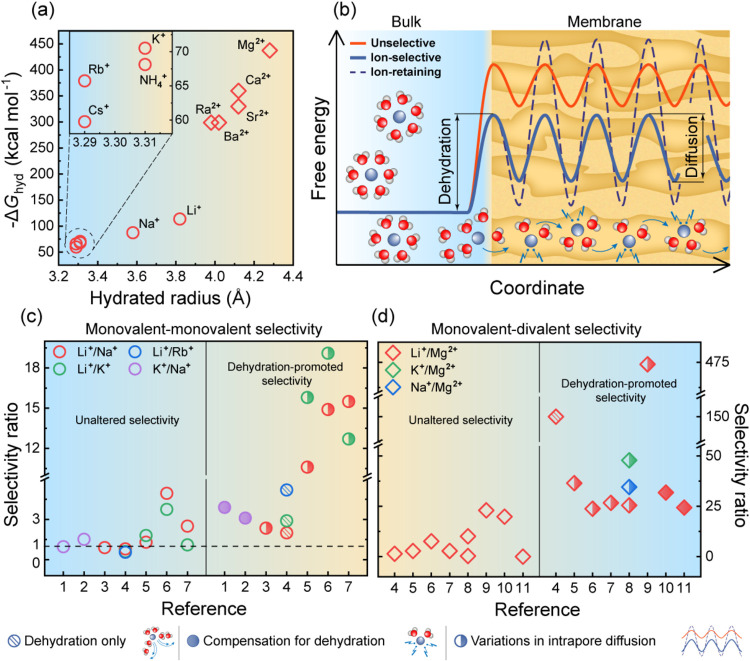
Dehydration-promoted selectivity in polymeric and composite membranes. (a) Relationship between hydrated radius^63^ and free energy of hydration (Δ*G*_hyd_)^137^ for monovalent and divalent cations. (b) Schematic representation of energy barrier profiles for ion transport across membranes under three different membrane design scenarios: (i) a conventional unselective membrane (orange); (ii) an ion-selective membrane (blue), which offsets the dehydration penalty and enables reversible compensation interactions to facilitate transport of the target ion; and (iii) an ion-retaining membrane (dashed), which offsets the dehydration penalty but imposes a significantly higher intrapore diffusion barrier due to strong interactions between the membrane interior and the target ion. (c) Selectivity ratio between monovalent ions (left panel—unaltered selectivity, right panel—dehydration-promoted selectivity). (d) Selectivity ratio between monovalent and divalent ions (left panel—unaltered selectivity, right panel—dehydration-promoted selectivity). References 1–11 in (c, d) correspond to the following sources:^83,93,138,140–146^ and,^147^ respectively.

The most prominent example of dehydration-promoted selectivity is provided by natural selective ion channels.^52,54,96,99,139^ The potassium channel is a noticeable example, where a narrow 12 Å long selectivity filter forces ions to shed their hydration shells, incurring an ion-specific energy penalty. Dehydrated potassium ions (K^+^) are compensated for this energy penalty through favorable, yet reversible interactions with precisely positioned binding sites along the filter. In contrast, smaller dehydrated sodium ions (Na^+^) have less energetically favorable interactions with these binding sites due to steric mismatch.^53,97,98^ This dehydration-promoted mechanism enables the potassium channel to achieve K^+^/Na^+^ selectivity ratios as high as 10^4^, far surpassing the selectivity observed in state-of-the-art polymeric membranes.^5^

The principles of dehydration-promoted selectivity can be applied to synthetic membranes by tuning the energy landscape for ion transport.^92,135,148^ This landscape, according to transport models of dense membranes, can be broken down into two steps, *i.e.*, partitioning and diffusion.^149,150^ Before an ion enters a membrane pore, it must partially or fully shed its hydration shell. The energy cost of this dehydration process, which typically increases with higher hydration strength of the ion and less favorable interactions between the partially dehydrated ion and the pore wall, largely determines the partitioning selectivity. Specifically, the ionic hydration strength and ability to favorably interact with the membrane are governed by the ionic intrinsic properties, such as charge, bare size, and geometry, which in turn dictate the hydrated size, hydration energy, and other properties. Once inside the pore, the partially dehydrated ion interacts with the membrane environment.^89–95^ The strength of these interactions dictates the diffusion barrier; ions that form stronger compensatory interactions with the pore walls may experience hindered transport (*i.e.*, higher diffusion barrier),^142,151–153^ whereas weakly interacting ions diffuse more freely (*i.e.*, lower diffusion barrier).

Due to this complex (and sometimes counteracting) effect of ion dehydration on the different transport steps, the impact of ion dehydration on ion–ion selectivity remains elusive (Figure 3b).^55^ Specifically, ions that more easily undergo dehydration and enter the membrane are expected to be more “sticky” to the pore wall;^154,155^ that is, while the partitioning of these ions into the membrane pore is relatively easy, their intrapore diffusion can be substantially hindered (Figure 3b, blue dashed line—ion-retaining energy profile).^153^ Compared to the ultrashort ion-selective biological channels, this counteracting effect becomes more prominent in thicker polymeric membranes where intrapore diffusion is often considered as the rate-limiting step of the transport,^5,156^ reducing the selectivity that is promoted by dehydration at the pore entrance. Improving this selectivity will therefore necessitate leveraging specific interactions that will be optimized to enhance dehydration of target ions at the pore mouth and minimize hindrance to the intrapore diffusion of these ions (Figure 3b, blue line—ion-selective energy profile).^138^

### Membrane Design for Dehydration-Promoted Selectivity

In recent years, significant progress has been made in enhancing ion–ion selectivity by leveraging specific interactions to promote the dehydration of target ions.^1,5,7,157–160^ Controlled dehydration is emphasized in the studies presented in Figure 3c,d as one of the key mechanisms enabled by specific membrane design strategies. One strategy for selectively promoting dehydration is to use subnanometer pores made of precisely controlled materials (*e.g.*, nanomaterial-assisted membranes,^161^ lamellar two-dimensional (2D) nanosheets with interlayer spacing,^141,144,145^ and porous framework materials).^83,138^ These well-controlled rigid structures impose steric hindrance, forcing ions to partially dehydrate in order to permeate. By tailoring the pore size to facilitate the transport of a target ion that has undergone partial dehydration while restricting others with higher hydration energies or hydrated sizes, a superior degree of selectivity can be achieved (Figure 3c,d; right panel).^162^ This approach of inducing controlled dehydration alone (without subsequent compensatory interactions) demonstrates the capability to significantly enhance ion separation (Figure 3c,d; hatched symbols).

Incorporating ion recognition sites within nanochannels can further refine selectivity by promoting specific binding interactions with partially dehydrated ions. This strategy is especially important in polymer membranes with less rigid and more flexible and amorphous pore structures. For example, integrating crown ethers into the membrane matrix introduces a mechanism of selective binding, as these macrocyclic molecules accommodate cations based on the size match between their cavity and the target ion.^142,163,164^ Specifically, crown ethers can partially compensate for the dehydration energy of the preferred ion at the membrane interface, facilitating its transport while largely rejecting other ions with stronger hydration and poor fit within the crown ether cavity (Figure 3c,d; filled symbols).^141,147^ Incorporation of certain chemical groups can also activate an opposite mechanism that favors the transport of a specific ion based on variations in intrapore diffusion (Figure 3c,d; half-filled symbols).^83,138,144,145^ For instance, sulfonate groups exhibit weaker binding affinity for Li^+^ compared to other monovalent ions (Na^+^ and K^+^) and especially divalent ions (Mg^2+^). This weaker interaction enables lithium to move more freely, effectively “hopping” between sulfonate sites, whereas the stronger binding of other ions impedes their transport.^145^

The combined effects of sterically induced ion dehydration and subsequent recognition within the pore offer a promising approach of designing highly selective membranes. However, precisely controlling these mechanisms at the angstrom-level remains challenging.^159,165^ In addition, fundamental gaps in understanding ion dehydration and transport under confinement persist, further complicating the synthesis of ion-specific membranes.^83,162^ The next section explores these challenges, emphasizing the need for improved fabrication techniques and advanced characterization methods.

## Outlook toward Ion-Specific Membranes

Although significant progress has been made, the development of membranes with high ion selectivity remains challenging due to the complexity of ion transport at the nanoscale. A key limitation lies in the speculative nature of dehydration mechanisms, where most insights are derived from simulations or indirect performance data rather than direct experimental validation.^35,41,115,166^ Additionally, practical challenges such as structural defects, requirements of angstrom precision, scalability limitations, and high fabrication costs continue to impede widespread application of dehydration-promoting membranes.^55,159^

Future efforts should focus on the development and implementation of defect-free and more scalable angstrom-level control over membrane structure and chemistry alongside postfabrication defect repair strategies^167^ and improved integration of selective layers. This may include further implementation of advanced materials like two-dimensional nanosheets, carbon nanotubes, and porous frameworks, which possess the potential for atomically tunable dimensions and homogeneous surfaces.^87,168^ Techniques such as atomic layer deposition enable precise tailoring of pore sizes and functionalities,^169,170^ while controlled incorporation of ion-recognition sites^171–175^ through prefunctionalized building blocks^176^ or postsynthetic modifications^158^ can enhance ion-specific interactions, mimicking the selectivity mechanisms of biological ion channels. Alternatively, techniques that enable the precise formation of subnanometer pores with functionalized interiors directly within robust polymer membranes, without relying on expensive nanomaterials, offer another promising direction. Specifically, incorporating specific additives in the classic interfacial polymerization reaction opens new possibilities for controlling ion permeability and regulating selectivity in polyamide membranes.^165^ This approach can minimize defect formation and provide a more practical pathway toward precise ion separation.

Achieving these advances in membrane design and fabrication, however, requires a deeper molecular-level understanding of dehydration-driven ion selectivity in confined membrane environments, an understanding that is currently limited by the capabilities of existing characterization techniques. For example, XAFS is a powerful, nondestructive technique that provides element-specific insights into ion coordination by analyzing the electronic structure, oxidation states, and ion-ligand interactions.^74,177–180^ While extensively used in bulk solution studies, idealized carbon nanochannels,^131^ and, to some extent, in polymeric membranes,^133^ XAFS is most effective for detecting local structures around ions with large atomic numbers, making it less suited for studying light ions like Li^+^ or Na^+^. In addition, its application to polymeric membranes requires further improvement due to challenges such as low signal-to-noise ratios, insufficient spatial and temporal resolution to resolve angstrom-scale confinement and capture rapid dehydration events, and difficulties in probing dynamic, heterogeneous polymer environments *in situ*.^76^ Overcoming these limitations and further expanding XAFS to membrane systems could offer new insights into ion hydration under confinement,^179,181^ aiding in the design of more selective membranes. Besides XAFS, NMR spectroscopy stands out as a versatile technique capable of providing valuable information on ion hydration, even for small ions like Na^+^ and F^–^, and has been successfully used to study dehydration and permeation in carbon nanopores.^132^ Furthermore, pulsed field gradient NMR (PFG-NMR) enables direct measurement of ion diffusion coefficients, providing a quantitative understanding of ion mobility within membrane pores.^75,182–184^ However, applying NMR and PFG-NMR to probe ion hydration and diffusion in heterogeneous polymeric membranes presents significant challenges, as internal magnetic field gradients from membrane matrix can distort signal attenuation, while rapid signal decay and low sensitivity for many common ions further complicate accurate data interpretation.^185^

Despite the potential of these advanced characterization techniques, ion dehydration in polymeric membranes remains largely unexplored. The disordered structure of polymeric membranes and the dynamic nature of ion hydration pose significant challenges for direct observation. As a result, fundamental aspects of ion dehydration, compensatory interactions, and selective transport mechanisms remain speculative and based on previous knowledge from biological channels. Expanding the use of these techniques and others (*e.g.*, Fourier-transform infrared spectroscopy, Raman spectroscopy)^76,186,187^ in polymeric membranes will be essential for advancing the field toward improved understanding of ion transmembrane permeation, validating theoretical models and refining membrane design strategies.

## Supplementary Material



## Notes

Charge, ionic radius, hydrated radius, coordination number, and hydration energy for different mono-, di-, and trivalent ions in the main group region of the periodic table (Table S1) (PDF)
