# Supplementary material for: Role of Ion Dehydration in Ion–Ion Selectivity of Dense Membranes
Source: Environ Sci Technol. 2025 Aug 19;59(34):17997–8009. doi: 10.1021/acs.est.5c04303 (PMC12409899; doi:10.1021/acs.est.5c04303)
Supplement: Supplementary file 1 [file es5c04303_si_001.pdf]

Supporting Information

Role of Ion Dehydration in Ion-Ion Selectivity of Dense Membranes

Alexander Ershov[1*], Hengyu Xu[2], Ying Li[2], Tiezheng Tong[3], Razi Epsztein[1*]

[1]Faculty of Civil and Environmental Engineering, Technion − Israel Institute of Technology, Haifa 32000, Israel

[2]Department of Mechanical Engineering, University of Wisconsin-Madison, Madison, WI, 53706, United States

[3]School of Sustainable Engineering and the Built Environment, Arizona State University, Tempe, AZ, 85287, United States

Contents:

1 Supporting Table

* Corresponding author Razi Epsztein. E-mail: raziepsztein@technion.ac.il; Tel: +972 (4) 829-3362

Corresponding author Alexander Ershov. E-mail: aleksandre@campus.technion.ac.il

Table S1. Charge, ionic radius, hydrated radius, coordination number, and hydration energy for different mono-, di-, and tri-valent ions in the main group region of the periodic table.

Ion	Charge	Ionic radius (nm)[a]	Hydrated radius (nm)[a]	Coordination number[b]	Hydration energy (kJ/mol)[c]
H_3O^+	1	0.115	0.282	4	-1050
Li^+	1	0.076	0.382	3-7	-475
Na^+	1	0.117	0.358	4-8	-365
K^+	1	0.149	0.331	4-8	-295
Rb^+	1	0.152	0.329	-	-275
Cs^+	1	0.186	0.329	5.3-8.2	-250
Be^{2+}	2	0.035	0.459	4	-2395
Mg^{2+}	2	0.072	0.428	6	-1830
Ca^{2+}	2	0.100	0.412	9.2-9.6	-1505
Sr^{2+}	2	0.116	0.412	9.8	-1380
Ba^{2+}	2	0.136	0.402	9.5	-1250
Ra^{2+}	2	-	0.398	-	-1250
Al^{3+}	3	0.500	0.475	6	-4525

[a] Data compiled from data in Ref. [1]

[b] Data from Ref. [2]

[c] Data summarized from Ref. [3]

REFERENCES

(1) Nightingale, E. R. Phenomenological Theory of Ion Solvation. Effective Radii of Hydrated Ions. *Journal of Physical Chemistry* 1959, *63* (9), 1381–1387. https://doi.org/10.1021/j150579a011.

(2) Ohtaki, H.; Radnai, T. Structure and Dynamics of Hydrated Ions. *Chem Rev* 1993, *93* (3), 1157–1204. https://doi.org/10.1021/cr00019a014.

(3) Marcus, Y. Thermodynamics of Solvation of Ions. *J.Chem.Soc., Faraday Trans.* 1991, *87* (18), 2995–2999.
